# In Vitro Secretome Analysis Suggests Differential Pathogenic Mechanisms between *Fusarium oxysporum* f. sp. *cubense* Race 1 and Race 4

**DOI:** 10.3390/biom11091353

**Published:** 2021-09-12

**Authors:** Yanqiu He, Xiaofan Zhou, Jieling Li, Huaping Li, Yunfeng Li, Yanfang Nie

**Affiliations:** 1 Guangdong Province Key Laboratory of Microbial Signals and Disease Control, South China Agricultural University, Guangzhou 510642, China; heyanqiuwork@sina.com (Y.H.); xiaofan_zhou@scau.edu.cn (X.Z.); Jielingbangbang@126.com (J.L.); huaping@scau.edu.cn (H.L.); 2 College of Plant Protection, South China Agricultural University, Guangzhou 510642, China; 3 College of Materials and Energy, South China Agricultural University, Guangzhou 510642, China

**Keywords:** *Fusarium oxysporum* f. sp. *cubense*, banana fusarium wilt, secretome, shotgun, effectors, bioinformatics

## Abstract

Banana Fusarium wilt, caused by the fungus pathogen *Fusarium oxysporum* f. sp. *cubense* (Foc), is a devastating disease that causes tremendous reductions in banana yield worldwide. Secreted proteins can act as pathogenicity factors and play important roles in the Foc–banana interactions. In this study, a shotgun-based proteomic approach was employed to characterize and compare the secretomes of Foc1 and Foc4 upon banana extract treatment, which detected 1183 Foc1 and 2450 Foc4 proteins. Comprehensive in silico analyses further identified 447 Foc1 and 433 Foc4 proteins in the classical and non-classical secretion pathways, while the remaining proteins might be secreted through currently unknown mechanisms. Further analyses showed that the secretomes of Foc1 and Foc4 are similar in their overall functional characteristics and share largely conserved repertoires of CAZymes and effectors. However, we also identified a number of potentially important pathogenicity factors that are differentially present in Foc1 and Foc4, which may contribute to their different pathogenicity against banana hosts. Furthermore, our quantitative PCR analysis revealed that genes encoding secreted pathogenicity factors differ significantly between Foc1 and Foc4 in their expression regulation in response to banana extract treatment. To our knowledge, this is the first experimental secretome analysis that focused on the pathogenicity mechanism in different Foc races. The results of this study provide useful resources for further exploration of the complicated pathogenicity mechanisms in Foc.

## 1. Introduction

Fusarium wilt disease, caused by *Fusarium oxysporum* f. sp. *cubense* (Foc), is one of the most destructive diseases in banana plants worldwide [1]. Fusarium wilt is a soil-borne disease that can destroy banana vascular bundles and cause plant death [2]. Foc has been classified into three physiological races according to their banana hosts, namely race 1 (Foc1), race 2 (Foc2), and race 4 (Foc4) [3]. Foc1 infects the cultivars ‘Gros Michel’ (AAA), ‘Pome’, ‘Silk’, and ‘Pisang Awak’ (ABB); Foc2 infects the cultivar ‘Bluggoe’ and its closely related cultivar; while Foc4 invades almost all banana varieties, including the Cavendish bananas (AAA) and the hosts of Foc1 and Foc2 [4]. Among these pathogens, Foc1 and Foc4 are widely distributed in China and significantly affect banana yield and quality [5]. Interestingly, Foc1 and Foc4 can invade some Cavendish cultivars (such as ‘Brazilian’) in common, but only Foc4 can cause plant diseases [6]. Recently, it has been shown in several studies that differences in pathogenicity between Foc1 and Foc4 are related to differences in the pectin methylesterases activity, oligogalacturonide contents, starch granules contents, and FA accumulation after pathogen infection in plants [6,7]. Dong et al. [8] suggested that ‘Brazilian’ differed in resistance to the two Foc races using a TMT-based comparative quantitative proteomics analysis. However, much still remains obscure on the mechanism of pathogenicity differences because of the complex genetic background of Foc [9,10].

Many phytopathogenic fungi secrete various extracellular proteins that perform diverse functions such as penetration, infection, colonization, expansion, nutrient acquisition, and protection against the host defense responses [11]. Especially, some secreted proteins can act as pathogenicity factors and play important roles in Foc–banana interaction during different infection stages [4,10,12]. For example, various cell wall-degrading enzymes, including pectin methylesterases, proteases, xylosidase, glucosidase, exopolygalacturonase, and xylanases are required for pathogenicity/virulence in Foc [5,6,13]. Foc also secretes many effectors (e.g., SIX, FTF1, OASTL) during host colonization to suppress or trigger plant immunity [14]. However, knowledge about Foc secreted proteins is very limited.

Over the last few years, the Foc–banana interactions have been studied at the molecular genetics, histological, infectious process, and proteome levels [8,15,16]. Proteomics is a powerful tool for studying the plant–fungus interaction mediated by such secreted proteins that facilitate the development of fungal diseases [17]. Characterization of the secretome of fungal pathogens would elucidate the pathogenic mechanisms used to infect, colonize, and invade their hosts [18]. With the completion of Foc genome sequences, bioinformatic approaches make possible the large-scale prediction and analysis of the entire set of secreted proteins in Foc. A previous genome-wide bioinformatic prediction of Foc secretome revealed 1000 putative secreted proteins [19]. A recent study has identified 919 non-redundant secreted proteins in Foc TR4, the *SGE1* and *FTF1* over-expression strains, of which 74 proteins were predicted to be candidate effectors using label-free quantitative proteomics approach [20]. Wang et al. [15] conducted a secretome analysis using HPLC-ESI-MS/MS and identified a total of 186 and 184 secreted proteins from Foc1 and Foc TR4 72 h by culturing Foc conidia alone or with banana roots, respectively. However, there still lacks a comprehensive experimental analysis of the Foc secretome. To the best of our knowledge, there has been no report of experimental secretome analysis that focused on the difference between Foc1 and Foc4.

In this paper, we analyzed the secreted protein profiles in Foc1 and Foc4 during spore germination by culturing Foc spore in banana extracts-containing medium to mimic the host–pathogen interaction. A shotgun-based proteomic approach was employed to identify the secreted proteins of Foc, followed by high-quality secretome prediction, in-depth in silico prediction and analysis, as well as RT-qPCR expression analysis of various pathogenicity factors. Our data, for the first time, provide a valuable resource for discovering the secreted proteins of Foc. Furthermore, the analysis of Foc secretome will also facilitate the understanding of the difference between Foc1 and Foc4 involved in the pathogenesis mechanisms. This study will also contribute to a better understanding of the molecular basis of Foc–banana interaction.

## 2. Materials and Methods

### 2.1. Chemicals

Aspartic acid, Tris, Phenylmethanesulfonyl fluoride (PMSF), Ethylene diamine tetraacetic acid (EDTA) and Albumin from bovine serum (BSA) were obtained from Bio-Rad (Hercules, CA, USA). Sequence-grade acetonitrile (ACN), trifluoroacetic acid (TFA), formic acid (FA), and acetone were purchased from Thermo Fisher Scientific (Waltham, MA, USA). Trypsin (sequencing grade) was from Promega (Madison, WI, USA). All remaining chemicals were purchased from Sigma-Aldrich (St. Louis, MO, USA) unless otherwise stated and were of analytical research grade. Milli-Q (Millipore, Billerica, MA, USA) water was used to make all solutions.

### 2.2. Plant Materials and Growth Conditions

The banana cultivar Brazilian (AAA group, Cavendish) was used in this study, which is susceptible to Foc4 but resistant to Foc1. Plants were maintained in a greenhouse at 25 ± 1 °C, 70–80% relative humidity with a 12-h photoperiod (250 μmol m^−2^ s^−1^). Banana seedlings at fully fourth-leaf stage were used for all experiments. Banana roots were washed with sterilized water to completely remove soil and stored at −80 °C for further use.

### 2.3. Fungi and Growth Conditions

Two Foc strains, Foc1 C2 and Foc4 DZ1, were used in this study. Both Foc isolates were confirmed their pathogenicity by inoculation onto their respective host banana cultivars in our previous study [13]. Foc mycelium were inoculated in Czapek Dox (CD) medium (0.3% *w*/*v* NaNO3, 0.1% *w*/*v* K_2_HPO_4_; 0.05% *w*/*v* KCl, 0.05% *w*/*v* MgSO_4_·7H_2_O, 3% *w/v* sucrose, pH 6.0) and cultured on a horizontal shaker at 28 °C for 4 days. Conidia were harvested by filtration through a 200 μm cell strainers, then centrifugated at 10,000× *g* for 15 min. Foc secretome in a given condition was prepared as described previously [21], with minor modifications. Briefly, the Foc conidia were inoculated into liquid NCM medium and NCMB medium to give a final concentration of 5 × 10^6^ conidia/mL and incubated at 28 °C in a rotary shaker at 120 rpm. NCM medium contains 1% *w*/*v* glucose, 0.4% *w*/*v* aspartic acid, 1× nitrate, 1× vitamins and 1× Trace element (pH 6.5). NCMB was the same as NCM, except for the medium addition of banana extracts. Briefly, banana roots were grounded thoroughly with liquid nitrogen. A dialysis bag (Sigma-Aldrich D0530, molecular weight cut-off of 12,400) enclosing 15 mL of NCM medium plus 15 mL of plant extract was then placed into 250 mL of NCM medium. A control medium only contained NCM medium which lacked a dialysis bag with plant extract. The cultures were collected at 7 h and 11 h post-inoculation for secreted protein preparation.

### 2.4. Extraction of Secreted Proteins

The secreted proteins were extracted, essentially as described [22] with some modifications. Briefly, the culture medium was filtered through 0.45 μm filter membrane (Millipore, Tullagreen, Ireland) and then sedimented by centrifugation for 10 min at 15,000× *g* to eliminate the germinating conidia and insoluble materials. The supernatants were mixed with PMSF (10 mM) and EDTA (5 mM) immediately and were used as crude protein solution. The supernatants were then concentrated by ultrafiltration using a PM-10 membrane (molecular weight cut-off of 10,000, Millipore, Tullagreen, Ireland) with 0.45 mPa N_2_ pressure. Three volumes of Tris-HCl (pH 7.5) were added to the residue solution and re-ultrafiltration three times. The final residue solution was further transferred to the Amicon Ultra-15 centrifugal filters (molecular weight cut-off of 3000, Millipore, Tullagreen, Ireland) and centrifuged at 18,000× *g* for 20 min. The supernatant was precipitated with acetone for 2 h at −20 °C, the precipitate was then collected by recentrifugation (18,000× *g*, 20 min) and dried by exposure to air. Finally, the precipitate was dissolved in a SDT lysis buffer (100 mM Tris-HCl, 4% *w*/*v* SDS, 1 mM DTT, pH 7.6) and stored at −80 °C until use. All the above procedures were carried out at 4 °C. Protein concentration was determined using the procedure described by Lowry, with BSA as the standard [23]. Each experiment was repeated three times by pooling independent germinating conidia cultural samples.

### 2.5. Identification of Proteins by LC-MS/MS

The secreted protein mixture of Foc1 and Foc4 was digested with trypsin using the FASP method [24], respectively. Briefly, approximately 200 μg proteins of Foc1 (or Foc4) were digested with 4 μg trypsin (Promega) in 40 μL 25 mM NH_4_HCO_3_ buffer overnight at 37 °C, and the resulting peptides were collected as a filtrate. The peptides of each sample were desalted on C18 Cartridges, concentrated by vacuum centrifugation and reconstituted in 40 µL of 0.1% *v*/*v* formic acid. The peptide mixture was loaded onto a reverse phase trap column (Thermo Scientific Acclaim PepMap100, 100 μm × 2 cm, nanoViper C18) connected to the C18-reverse phase analytical column (Thermo Scientific Easy Column, 10 cm long, 75 μm inner diameter, 3 μm resin) in buffer A (0.1% Formic acid) and separated with a linear gradient of buffer B (84% acetonitrile and 0.1% Formic acid) at a flow rate of 300 nl/min controlled by IntelliFlow technology. LC-MS/MS analysis was performed on a Q Exactive mass spectrometer (Thermo Scientific, Wilmington, DE, USA) that was coupled to Easy nLC (Thermo Fisher Scientific, Waltham, MA, USA) for 60 min. The mass spectrometer was operated in positive ion mode. MS data were acquired using a data-dependent top10 method dynamically choosing the most abundant precursor ions from the survey scan (300–1800 *m*/*z*) for HCD fragmentation. The MS data were searched using MASCOT engine (Matrix Science, London, UK; version 2.4) against the UniProtKB *Fusarium oxysporum* f. sp. *cubense* database. For protein identification, the following options were used: trypsin cleavage, peptide mass tolerance set to 20 ppm, MS/MS tolerance set to 0.1 Da, missed cleavage set to 2, carbamidomethylation set as fixed modification, FDR ≤ 0.01.

### 2.6. Bioinformatics Analyses of the Secreted Proteins

The N-terminal signal peptides in the secreted proteins were detected by using SignalP 6.0 [25]. Transmembrane domains in proteins were predicted with Phobius 1.01 [26] and TMHMM 2.0 [27]. Glycosylphosphatidylinositol (GPI) anchor site was predicted using the Big-PI Fungal Predictor server (https://mendel.imp.ac.at/gpi/fungi_server.html, last accessed: 16 August 2021) [28] and the PredGPI prediction server (http://gpcr2.biocomp.unibo.it/predgpi/, last accessed: 16 August 2021) with default parameters [29]. Endoplasmic reticulum (ER) retention signal in proteins was detected by using ScanProsite to scan against the PROSITE motif PS00014 for ER targeting sequence [30]. Subcellular localization of proteins was predicted by DeepLoc 1.0 [31], ProtComp 9.0 (standalone version obtained from http://linux5.softberry.com/cgi-bin/download.pl?file = protcompan, last accessed: 16 August 2021), TargetP 2.0 [32], and WoLF PSORT 0.2 [33].

### 2.7. Functional Annotation of Secreted Proteins

EggNOG-mapper v2.1.4 was used to obtain a rich set of functional annotations for proteins, including COG functional categories, Gene Ontology terms, KEGG Orthology assignments, protein domains, and functional descriptions [34].

### 2.8. Prediction of Pathogenicity-Associated Secreted Proteins

Carbohydrate active enzymes (CAZymes) were identified and classified by the dbCAN2 meta server (http://bcb.unl.edu/dbCAN2/; dbCAN HMMdb v9, last accessed: 16 August 2021) using all the three available tools, including HMMER (*e*-value < 1 × 10^−15^, coverage > 0.35), DIAMOND (*e*-value < 1 × 10^−102^), and Hotpep (frequency > 2.6, hits > 6) [35]. Only the proteins successfully annotated by at least two of the tools were considered CAZymes Putative virulence proteins were predicted by searching Foc secreted proteins against the PHI-base database using BLASTP (*e*-value < 1 × 10^−5^) [36].

### 2.9. Prediction of Effectors

Three independent approaches were used to identify candidate effectors from Foc secretome in this study: (1) the machine learning method EffectorP 3.0 [37] was used for effector prediction; (2) proteins that are small in size (≤400 amino acids) and rich in cysteine residues (≥4) were identified and considered candidate effectors [38]; and (3) proteins that match with known effectors in the PHI-base database were also considered effectors. The final set of candidate effectors was a union of the results of the three approaches.

### 2.10. Quantitative Real-Time PCR (RT-qPCR) Analysis

Total RNA was extracted from Foc using a Fungal RNA kit (Omega Bio-tek, Norcross, GA, USA) according to the manufacturer’s protocol. RNA was reversely transcripted in 20 μL of reaction system using the PrimeScript TM RT Master Mix Kit (TaKaRa, Beijing, China) following the manufacturer’s instructions. Gene-specific primers for RT-qPCR (Supplemental Table S1) were designed using Primer 5.0 software. The RT-qPCR was conducted on a CFX Coxnnect^TM^ Real-Time System (Bio-Rad, Hercules, CA, USA) with the SYBR Premix Ex Taq Kit (TaKaRa, Beijing, China) according to the manufacturer’s instructions. The tublin gene was used as a reference. Each sample was represented by three biological replicates. Relative transcript levels for each gene were calculated using the formula 2^−ΔΔCT^ [39].

### 2.11. Statistical Analysis

Statistical analyses were carried out using ANOVA by the statistical program SPSS 13.0 for Windows (SPSS Inc., Chicago, IL, USA). Multiple differences among means were evaluated using Duncan’s multiple range tests at a 5% probability level. To determine the significant difference among group means, the repeated measurement was given as means ± standard error (SE). Supplemental figures and supplemental tables contain detailed information of all supplemental files to support this study.

## 3. Results

### 3.1. Shotgun Proteomic Analysis of Foc Secretomes

The aim of this work was to analyze and compare the secretomes of Foc1 and Foc4 using a shotgun-based proteomic approach in order to better understand the difference in pathogenicity between Foc1 and Foc4. Two distinct stages of Foc conidial germination were chosen for secretome analysis, corresponding to germination tube elongation from conidia (7 h) and visible full mature mycelia (11 h), respectively [40]. To mimic the Foc–banana interaction and maximize the number of secreted proteins, we germinated conidia at a high concentration in a NCM medium plus a dialysis bag enclosing 50% banana root extract. A total of 350 ± 36 μg secreted proteins of each treatment were obtained from 1 L of culture medium in this study. To test the efficiency and reproducibility of the secreted proteins of Foc1 and Foc4, total proteins were also submitted to SDS-PAGE with loading amounts of about 10 μg per sample. The representative gel was shown in Supplemental Figure S2. CBB staining also showed that similar bands of the secreted proteins of Foc1 and Foc4 were reproducibly detected on the gel.

To study Foc secretomes, we combined the proteins collected at 7 h and 11 h as one sample for each of Foc1 and Foc4, respectively. For each Foc strain, three independent mixed samples were analyzed by LC-MS/MS. In total, 1183 and 2450 non-redundant proteins were identified in Foc1 and Foc4, respectively. Most of the proteins were consistently detected in all three biological replicates (73.5% in Foc1 and 74.5% in Foc4), indicating high reproducibility of our analysis (Supplemental Figure S2). The length of the proteins ranged from 65 to 5579 amino acids (aa) in Foc1 and from 51 to 6825 aa in Foc4, with the medium length being 380 aa in Foc1 and 412 aa in Foc4 (Supplemental Figure S3). There are 819 common proteins shared by Foc1 and Foc4 (Supplemental Figure S4).

### 3.2. In silico Analysis of Foc Secretomes

**Figure 1:**
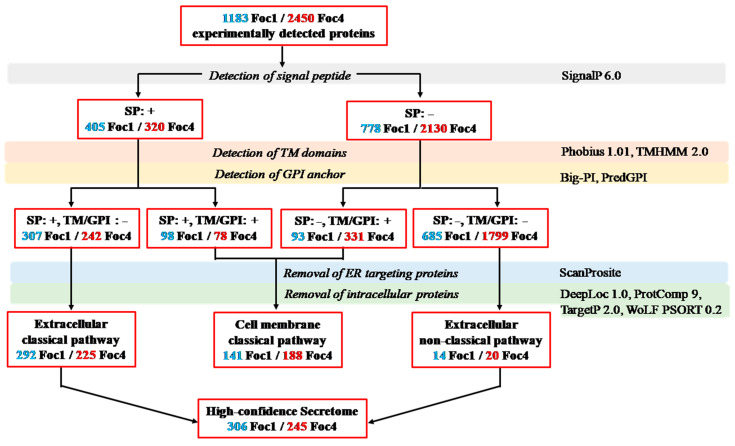
The bioinformatics pipelines used to predict the Foc1 and Foc4 secretomes.

In silico analysis of Foc secretomes was performed using a collection of ten state-of-the-art protein localization prediction tools. The bioinformatics workflow used to predict and classify secreted proteins was shown in Figure 1 (all the analysis results were available in Supplementary Table S2). Firstly, all 1183 Foc1 and 2450 Foc4 proteins identified in our proteomic analysis were examined for the presence of signal peptide (SP; by SignalP 6.0), transmembrane (TM) domain (by Phobius 1.01 and TMHMM 2.0), Glycosylphosphatidylinositol (GPI) anchor (by Big-PI and PredGPI). Based on the results, the proteins were separated into three categories, including (I) 307 Foc1 and 242 Foc4 proteins that contain a SP but lack TM and GPI; (II) 191 Foc1 and 409 Foc4 proteins that contain TM domain(s) and/or GPI anchor(s), while they may or may not have a SP; and (III) 685 Foc1 and 1799 Foc4 proteins that have neither of the three sequence features. Subsequently, the proteins were screened by ScanProsite to detect endoplasmic reticulum (ER) retention signal. Additionally, subcellular localizations of the proteins were predicted by TargetP 2.0, DeepLoc 1.0, ProtComp 9.0, and WoLF PSort 0.2 in combination. Proteins that contain ER retention signal or are predicted to be intracellular were removed from each category. Finally, the 292 Foc1 and 225 Foc4 proteins remained in category I (SP: +, TM/GPI: −) were classified as “extracellular” proteins secreted via the classical pathway, the 141 Foc1 and 188 Foc4 proteins remained in category II (SP: +/−, TM/GPI: +) were classified as “cell membrane” proteins secreted via the classical pathway, while the 14 Foc1 and 20 Foc4 proteins remained in the category III (SP: −, TM/GPI: −) were classified as “extracellular” proteins secreted through the non-classical pathway. Notably, 182 extracellular and 82 cell membrane proteins in the classical secretion pathway, as well as 9 extracellular proteins in the non-classical secretion pathway are shared by Foc1 and Foc4 (Supplementary Figure S4). Taken altogether, 447 Foc1 and 433 Foc4 secreted proteins were predicted by our in silico analysis, accounting for 37.79% and 17.67% of all proteins experimentally detected in the two strains, respectively. In each race, the “extracellular” classical and non-classical secreted proteins were combined and hereafter referred to as the “high-confidence secretome” (with 306 proteins in Foc1, and 245 proteins in Foc4), which was our focus in all downstream analyses.

### 3.3. Functional Annotation and Classification of the Secreted Proteins

**Figure 2:**
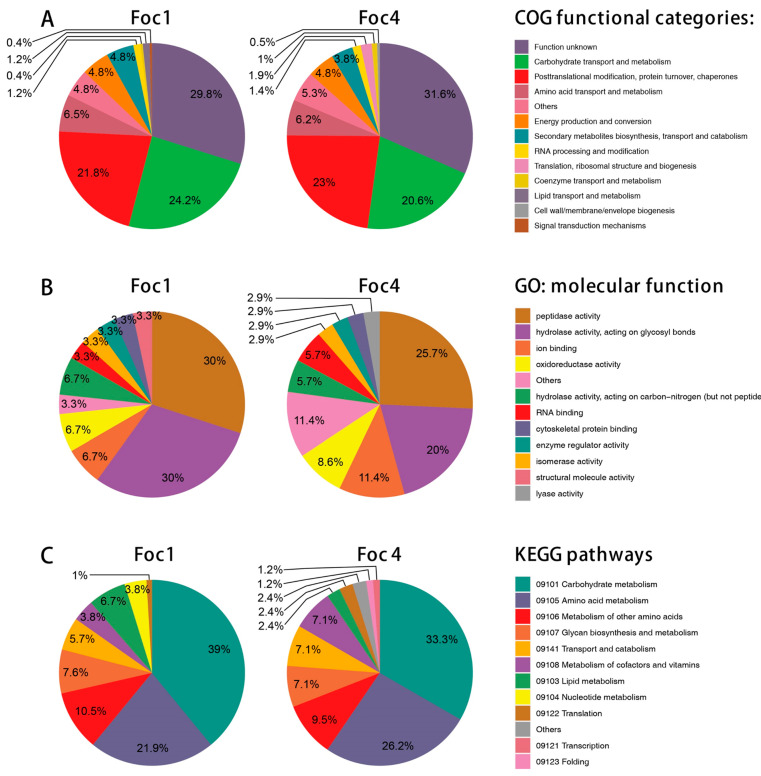
Functional annotations of the high confidence secretomes of Foc1 and Foc4. (**A**) COG functional categories; (**B**) GO molecular function terms; (**C**) KEGG pathways. The top 10 items in each type of functional annotations were shown.

Functional annotation of the Foc1 and Foc4 high-confidence secretomes was carried out using EggNOG-mapper. In total, 249 of the 306 (81.37%) Foc1 secreted proteins and 210 of the 245 (85.71%) Foc4 secreted proteins were assigned with one or more COG/GO/KEGG terms (Supplementary Table S2). In the secretomes of both races, the most abundant COG functional category was S (“function unknown”) which covers 74 proteins (24.18%) in Foc1 and 66 proteins (26.94%) in Foc4 (Figure 2A). Taking the proteins without any functional annotation into account, this result indicates that a considerable fraction of the Foc1 and Foc4 secreted proteins remain functionally uncharacterized. The next two most abundant categories in both races were G (“carbohydrate transport and metabolism”), which contains 60 (19.61%) Foc1 and 43 (17.55%) Foc4 proteins, and O (“posttranslational modification, protein turnover, chaperones”), which contains 57 (18.63%) Foc1 and 51 (20.82%) Foc4 proteins (Figure 2A). Most proteins in the G category were also annotated as CAZymes (see next section), which is consistent with their critical roles in pathogen-host interaction. The category O, on the other hand, contains many peptidases, suggesting that they might also be important for the pathogenicity of Foc. In comparison, the other functional categories have much fewer proteins, but their relative abundances were still similar between Foc1 and Foc4. Similar trends were revealed in the analyses of GO (Figure 2B) and KEGG annotations (Figure 2C).

### 3.4. CAZymes Analysis of the Secreted Proteins

**Figure 3:**
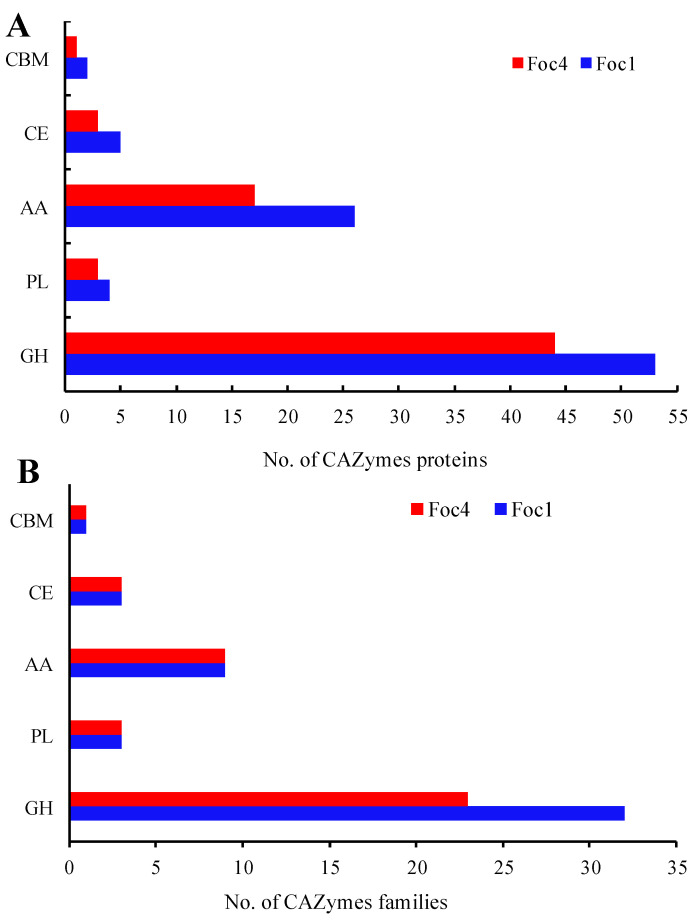
Comparison of CAZymes proteins (**A**) and CAZymes families (**B**) identified in Foc1 and Foc4 secretomes.

The Foc1 and Foc4 secretomes were also annotated with the CAZy database and identified CAZymes were further assigned to CAZy families in the enzyme classes of glycoside hydrolases (GHs), glycosyl transferases (GTs), polysaccharide lyases (PLs), carbohydrate esterases (CEs), auxiliary activities (AAs), and carbohydrate-binding modules (CBMs). In total, 90 Foc1 and 68 Foc4 CAZymes were identified, accounting for 29.41% of the 306 and 27.76% of the 245 proteins in Foc1 and Foc4 secretomes, respectively (Supplementary Table S2). GH was the most abundant enzyme class in both strains, containing 53 Foc1 proteins in 32 families, and 44 Foc4 proteins in 23 families. The second most abundant class was AA, containing 26 Foc1 proteins in 9 families and 17 Foc4 proteins in 9 families. In comparison, the classes CE, CBM, and PL were much smaller and contained up to five proteins in each strain. On the other hand, the GT class was missing entirely in Foc1 and Foc4 secretomes (Figure 3, Supplementary Table S2).

**Figure 4:**
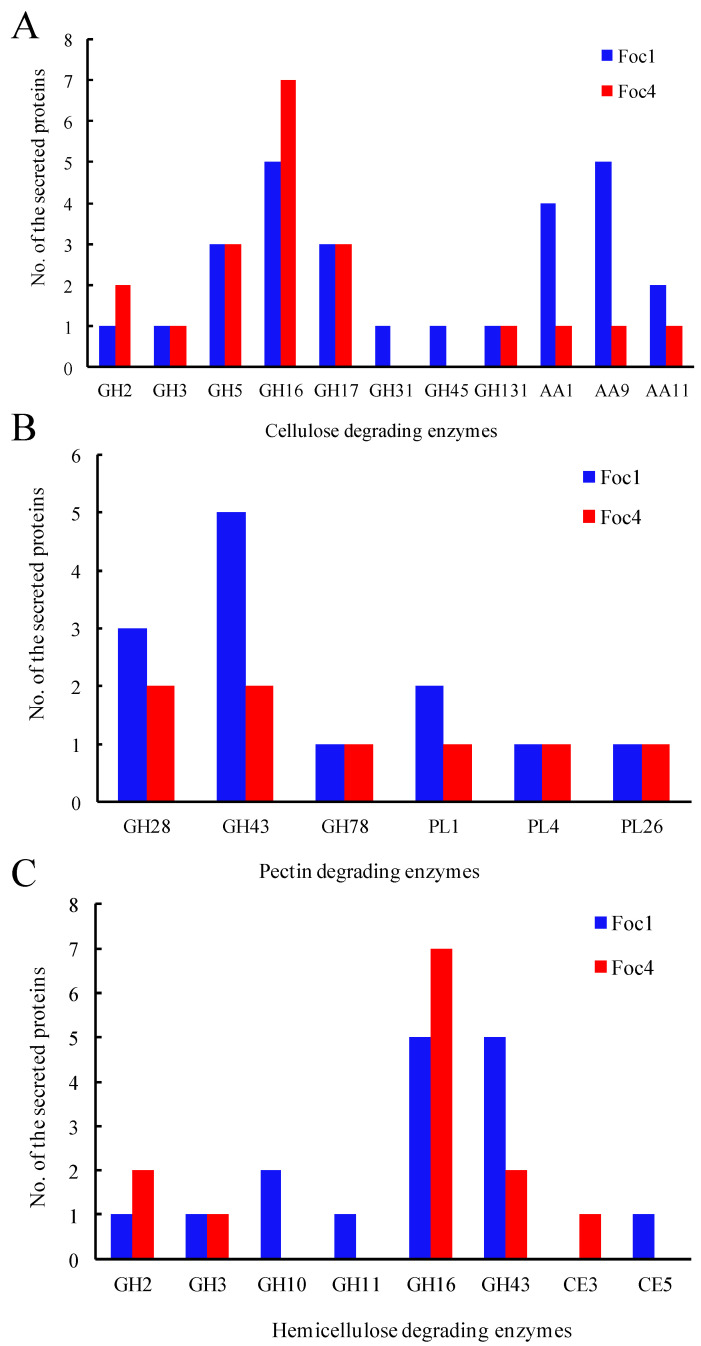
Comparison of cell wall-degrading enzymes between Foc1 and Foc4. (**A**), cellulose-degrading enzymes; (**B**), pectin degrading enzymes; (**C**), xylan degrading enzymes.

Many CAZymes are known as cell wall-degrading enzymes (CWDEs) due to their important roles in the degradation of plant cell wall, which have been demonstrated to be associated with pathogenicity or virulence [41,42]. In this study, we identified 42 and 36 CWDE-related proteins in Foc1 and Foc4, respectively (Figure 4; Supplemental Table S2). Specifically, 27 Foc1 and 20 Foc4 proteins were identified as cellulose-degrading enzymes (Figure 4A), 13 Foc1 and 8 Foc4 proteins were identified as pectin degrading enzymes (Figure 4B), while 16 Foc1 and 13 Foc4 proteins were identified as hemicellulose-degrading enzymes (Figure 4C). Overall, our inter-specific comparisons revealed largely similar repertoires of CAZymes in the two Foc races, including the proteins involved in degrading cellulose, hemi-cellulose and pectin of plant cell walls.

### 3.5. Pathogenicity-Associated Secreted Proteins

Foc1 and Foc4 secreted extracellular proteins were also annotated with the PHI-base database, which contains expert-curated information on experimentally verified pathogenicity, virulence, and effector genes from phytopathogenic fungi and other eukaryotic and prokaryotic pathogens. A total of 159 Foc1 and 130 Foc4 proteins were found to match with sequences in PHI-base, accounting for 51.96% and 53.06% and the Foc1 and Foc4 secretomes, respectively (Supplementary Table S2). Most of these proteins were predicted to have pathogenicity-related phenotypic outcomes in mutation experiments. Specifically, 85 Foc1 and 76 Foc4 proteins were associated with “reduced virulence”, among which 59 were present in the secretomes of both strains. In addition, three Foc4 proteins were associated with “loss of pathogenicity”, whereas two Foc1 and one Foc4 proteins were annotated as “increased virulence” (Table 1); interestingly, all of these proteins were unique to either secretome.

### 3.6. Effector Analysis of Foc Secretome

Effector candidates in Foc1 and Foc4 secretomes were identified using three independent approaches, including: (1) 74 Foc1 and 58 Foc4 proteins were predicted to be fungal effectors by EffectorP 3.0; (2) 13 Foc1 and 10 Foc4 proteins were classified as small secreted cysteine-rich proteins (SSPs) based on their protein length (≤400 aa) and the number of cysteine residues (≥4) [43]; and (3) seven Foc1 and three Foc4 proteins were annotated as “effector” in the above-mentioned PHI-base analysis. Altogether, 87 Foc1 and 70 Foc4 effectors were predicted in at least one of the three analyses, and 53 of these effectors were present in the secretomes of both strains (Supplementary Table S2).

### 3.7. RT-qPCR Analysis of Foc Secreted Proteins

**Figure 5:**
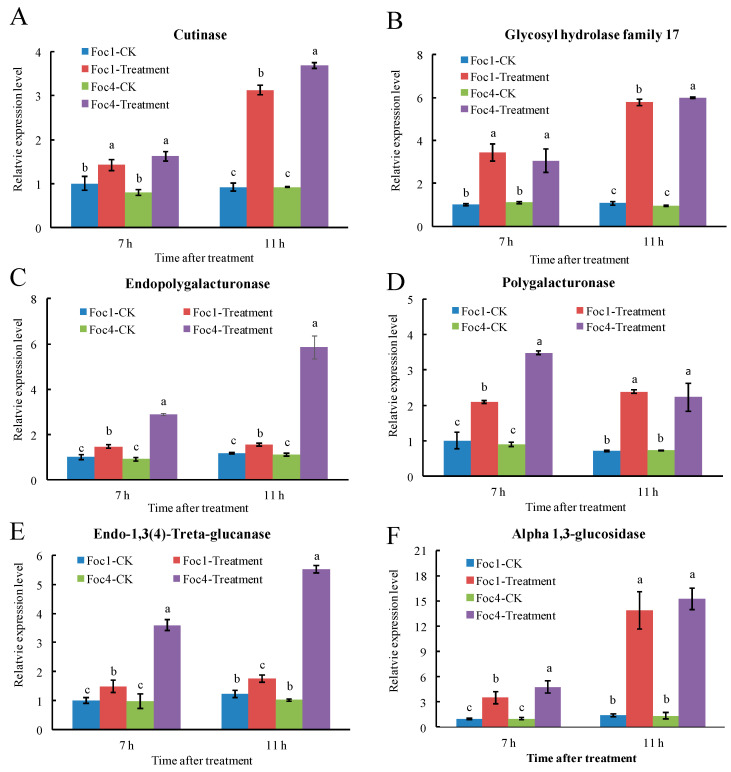

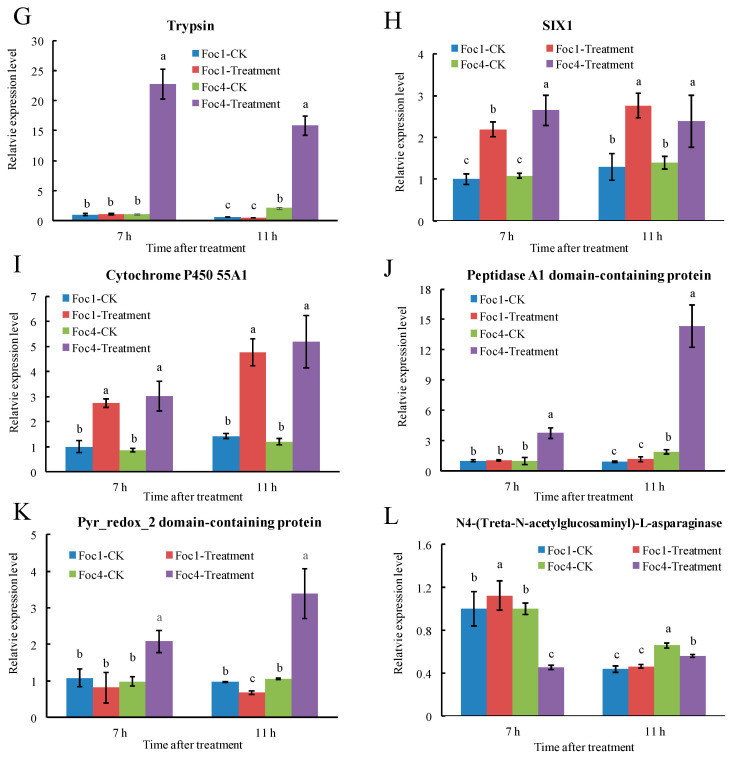
Expression analysis by RT-qPCR of 12 secreted protein genes after banana plant extracts treatment. (**A**): cutinase; (**B**): glycosyl hydrolase family 17; (**C**): endopolygalacturonase; (**D**): polygalacturonase; (**E**): endo-1,3(4)-Treta-glucanase; (**F**): alpha 1,3-glucosidase; (**G**): trypsin; (**H**): SIX1; (**I**): cytochrome P450 55A1; (**J**): peptidase A1 domain-containing protein; (**K**): Pyr_redox_2 domain-containing protein; (**L**): N4-(Treta-N-acetylglucosaminyl)-L-asparaginase. Values are the means (±SE) based on three independent experiments, and bars indicate standard deviations. Different small letters in each group indicate significant differences at *p* ≤ 0.05.

We carried out expression analysis via RT-qPCR on twelve genes encoding various CWDEs and pathogenicity factors (see sequence accessions in Supplemental Table S3), including cutinase, glycosyl hydrolase family 17, endopolygalacturonase, polygalacturonase, endo-1,3(4)-Treta-glucanase, alpha 1,3-glucosidase, trypsin, SIX1, cytochrome P450 55A1, peptidase A1 domain-containing protein, Pyr_redox_2 domain-containing protein, and N4-(Treta-N-acetylglucosaminyl)-L-asparaginase (Figure 5). The expression levels of these genes were measured in both Foc1 and Foc4 at two time points (7 h and 11 h) after induction by banana extract. Our results showed that the expression levels of the gene encoding CWDEs, including cutinase, glycosyl hydrolase family 17, endopolygalacturonase, polygalacturonase, endo-1,3(4)-beta-glucanase, and alpha 1,3-glucosidase were increased significantly after induction by banana extracts in both Foc1 and Foc4 (Figure 5A–F). Similarly, the expression levels of genes encoding secreted in xylem 1 (SIX1) (Figure 5H) and cytochrome P450 (Figure 5I) were also increased significantly in both Foc1 and Foc4. In contrast, genes encoding two pathogenicity factors, namely trypsin and peptidase A1 domain-containing protein, showed significantly increased expression in Foc4 but not in Foc1 (Figure 5G,J). The Pyr_redox_2 domain-containing protein-coding gene showed a significant decrease in its expression only in Foc4 (Figure 5K). The gene encoding N4-(Beta-N-acetylglucosaminyl)- L-asparaginase showed significantly increased expression in Foc1 but decreased expression in Foc4 (Figure 5L). Taken together, our data showed that the expression of CWDEs can be induced by banana extracts both in Foc1 and Foc4, whereas some pathogenicity-factor encoding genes were only induced in Foc4, which mirrors the fact that Foc1 and Foc4 can invade successfully ‘Brazilian’, but only Foc4 can cause plant diseases. The results highlighted the complex pathogenicity mechanism at multiple molecular levels in Foc1 and Foc4.

### 3.8. Functional Characteristics of Other Experimentally Detected Proteins

In this study, a total of 1183 Foc1 and 2450 Foc4 proteins were detected in our proteomic assay. So far, we have focused our analyses on the proteins classified in the classical and non-classical secretion pathway, which represent a high confidence set of secreted proteins. At the same time, the remaining 736 Foc1 and 2017 Foc4 proteins were also experimentally detected (Supplementary Table S2) and might also contain important pathogenicity-related factors that were secreted through currently uncharacterized mechanisms. Therefore, we also carried out functional annotation of these proteins (Supplementary Table S2) and found that: (1) 22 Foc1 and 29 Foc4 proteins were annotated as CAZymes; (2) 348 Foc1 and 951 Foc4 proteins had significant matches with sequences in PHI-base, among which 205 and 549 proteins were associated with altered virulence in mutation experiments; and (3) 265 Foc1 and 659 Foc4 proteins were predicted to be effectors.

## 4. Discussion

During fungi–plant interaction, fungi can secrete a large number of proteins to manipulate the immunity and physiology of their hosts to escape host recognition, suppress plant defenses, facilitate infection, and/or induce plant cell death [44,45]. Analysis of the secretome is a powerful tool to investigate how fungi manage the infection process [46]. In our previous studies, 5989 secreted proteins, including 988 classically secreted proteins and 5001 SecretomeP-predicted non-classically secreted proteins, were detected in Foc1 by genome-scale prediction [47], while 10,270 secreted proteins, including 1054 classically secreted proteins and 9216 non-classically secreted proteins, were detected in Foc4, representing 38.8% and 45.7% of the respective genomes [48]. However, it is a challenging task to accurately predict fungal secreted proteins based on computational methods alone, particularly for those that lack the signal peptide and are thus secreted through non-classical secretory mechanisms. Therefore, despite their importance, such computational predictions do not represent reality as many postulated genes do not have transcriptional or translational functions [49,50]. Therefore, direct experimental proof of protein secretion is needed and critical.

In this study, we employed a shotgun-based proteomic approach to identify secreted proteins of Foc1 and Foc4 in an in vitro experiment setup, in which the pathogens were induced by banana root extracts to mimic the early growth and development of Foc during initial infection processes. In this in vitro secretome analysis, a total of 1183 non-redundant proteins in Foc1 and 2450 non-redundant proteins in Foc4 were identified, representing only 19.8% and 23.9% of the above-mentioned genome-scale predicted secretomes of Foc1 and Foc4, respectively. It should be noted that our shotgun proteomic analyses were highly reproducible, and similar numbers (c.a. 1500) of proteins were detected in a recent proteomic study of the secretomes of two pathogenic fungi [51], suggesting that our experimental results are reliable. The drastic discrepancy between previous genome-scale predictions and our experimental results here might have the following potential explanations: (1) the interaction between Foc and banana is a highly complex process and the pathogens are able to modulate their secretomes in response to their plant hosts, yet the in vitro banana–Foc interaction model used in this study is a simplification and thus might not fully capture the real interaction mechanism; (2) previous studies have shown that the actual composition of the secretome might vary greatly under different growth conditions, and thus, it is very likely that the conidia samples obtained at 7 h and 11 h only provide partial coverage of the whole secretome profiles of Foc; (3) the abundance of some secreted proteins might be too low to be detected by the instrument because of their limited sensitivity and resolution [52]; and (4) the Foc secretomes might be over-estimated in previous genome-scale predictions, particularly the large number of proteins in the non-classical secretion pathway predicted by SecretomeP which was originally designed for mammals and has been shown to perform poorly on other eukaryotes. The difference might actually be due to a combination of some or all of the above biological and analytical factors. Nonetheless, the composition and dynamics of Foc secretomes are still not completely revealed and further experimental investigations are needed.

Among plant pathogens, necrotrophic fungi secrete larger numbers of proteins than hemibiotrophic and biotrophic fungi [53]. In general, most of the proteins are secreted outside the cell through the conventional Golgi/ER secretory pathway [54]. However, protein secretion mechanisms in fungi still remain poorly understood. For instance, recent work have disclosed a new type of secreted proteins, referred to as leaderless secretory proteins (LSPs), that were secreted through the unconventional secretory pathways in fungi [50]. Interestingly, these LSPs account for more than 50% of the total identified secretome in some fungi [53]. In this study, 252 proteins in Foc1 and 614 proteins in Foc4 were identified as LSPs, representing 21.3% and 25.1% of the identified proteins, respectively. However, 54.9% (650 proteins) in Foc1 and 66.2% (1626 proteins) in Foc4 of the remaining identified proteins could still not be predicted to be secreted proteins by these bioinformatics programs. Previous studies also showed that 17.6% of *Magnaporhe oryzae* secretome could not be predicted to be secreted proteins by a number of bioinformatics tools [55]. These results suggested that Foc may possess yet unknown secretory mechanisms in addition to well-characterized Golgi/ER, or unconventional (independent of Golgi/ER) secretory mechanisms. In this work, most of the proteins experimentally detected in our proteomic analysis were not predicted in either classical or non-classical secretion pathways, suggesting that they might be secreted through currently unknown mechanisms. Importantly, we have also predicted a wealth of potentially important pathogenicity factors among them, providing a valuable resource for further investigation.

Fungal pathogens, especially necrotrophic pathogens, produce a variety of CAZymes for the degradation of plant polysaccharide materials to facilitate infection and/or gain nutrition [56,57,58,59]. CAZymes that are known as plant cell wall degradation enzymes mainly include pectinases, cellulases, and hemicellulase, which can destroy plant epidermis and act as important virulence factors during the initial infection process [60,61]. In this study, we found that the numbers and types of CAZymes in Foc1 and Foc4 were relatively similar, which is consistent with previous reports that both Foc1 and Foc4 can successfully invade banana plants [5,62]. Therefore, the similar repertoires of CAZymes may be an important reason for the successful infection of both Foc1 and Foc4 on Brazilian plants.

PHI-base database can be used to find novel pathogenic genes in pathogenic fungi [63]. Increasing evidence has shown that virulence-related proteins play a pivotal role in the process of fungal pathogens against plant defense. Previous studies showed that some well-characterized virulence-related proteins in *Fusarium* were found in the search of PHI database, such as ATG15 [64], NPC1 [65], MCC [66], FOW [67], CHS [68], FGA [69], and FGB [70]. In this study, we identified 159 and 130 putative virulence-associated proteins in Foc1 and Foc4, respectively. The results were also consistent with the previous study [19], indicating the critical role of the virulence-related proteins which mediated Foc to infect ‘Brazilian’. Interestingly, some virulence-related proteins were only found in Foc4, such as FOW, FGA, CHS and FGB. Thus, it would be of interest to explore how Foc4 utilizes this arsenal of putative virulence proteins for its survival and host infection, whereas Foc1 lacks pathogenicity during host penetration.

Effectors are key pathogenic factors of fungi to facilitate infection or trigger defense responses on host plants [53,71,72]. In previous studies, many effectors had been reported in *Fusarium oxysporum*, including secreted in xylem (SIX) [73], Necrosis proteins (NPP1) [74], Cerato-Platanin [75], hydrophobins [76], and LysM effectors [77]. In this study, a total of 87 and 70 effectors were predicted in Foc1 and Foc4, respectively, among which many well-known effectors were commonly found in both races, such as SIX1, SIX6, SIX9, LysM, Cerato-platanin, and NPP1. However, the set of candidate effectors also included numerous secreted proteins without any recognizable Pfam domain or functional annotation, which may represent novel effectors in Foc. In previous studies, this type of candidate effectors was also found in some fungi and was thought to play a crucial role in enabling fungal colonization of plant tissue [78,79]. However, little is known about the functions of these pathogenic factors and further investigations are needed. Interestingly, two and five Foc4 unique effectors were annotated as “loss of pathogenicity” and “reduced virulence”, respectively, in our PHI-base analysis, suggesting that they likely have important roles in the pathogenicity of Foc4. Similarly, six Foc1 unique candidate effectors were annotated with the “reduced virulence” phenotype. Furthermore, our expression analysis showed that, for the pathogenic factor encoding genes shared by both races, their expression regulation during infection might be substantially different between Foc1 and Foc4. Taken together, we speculate that the pathogenic difference between Foc1 and Foc4 may be partly attributed to the differences in the composition and expression of their candidate effectors.

## 5. Conclusions

In this study, we conducted a comparative proteomic analysis of the secretomes of Foc1 and Foc4, in order to better understand their differential pathogenic mechanisms. A total of 1183 and 2450 non-redundant secreted proteins were identified in Foc1 and Foc4, respectively, and were further classified into classical and non-classical secretion pathways, as well as proteins that might be secreted through currently unknown mechanisms, which enriches our understanding of how Foc orchestrates the secretion of proteins during its early growth and infection processes. Through functional annotation and comparison of the secretomes of Foc1 and Foc4, we found that the repertoires of CAZymes were highly similar between them. However, the two races exhibited significant differences in secreted proteins involved in virulence, pathogenicity, and effectors. which might explain why Foc1 and Foc4 could successfully invade the host plants but showed different pathogenicity against banana host. Moreover, quantitative PCR analysis showed that the expression of several genes encoding secreted pathogenicity factors changed significantly in response to the induction of banana extracts. Future functional investigation of these pathogenicity-related secreted proteins, many of which have unknown functions, might provide new insight into Foc pathogenicity and how to control infection at the early stages. Overall, this study provides useful clues for further exploration of the complicated pathogenicity mechanisms in Foc.

## Figures and Tables

**Table 1:** Predicted PHI proteins of the secreted proteins of Foc1 and Foc4.

Phenotype	No. of PHI-Base Matches		Fraction of the Secretome (%)	
	Foc1	Foc4	Foc1	Foc4
Loss of pathogenicity	0	3	0	1.22
Reduced virulence	85	76	27.78	31.02
Unaffected pathogenicity	53	35	17.32	14.29
Effector (plant avirulence determinant)	7	3	2.29	1.22
Increased virulence	2	1	0.65	0.41
Lethal	1	1	0.33	0.41
Mixed outcome	11	11	3.59	4.49

## Data Availability

The data presented in this study are available in Supplementary Figures S1–S4 and Supplementary Tables S1 and S2.

## Supplementary Materials

The following are available online at https://www.mdpi.com/article/10.3390/biom11091353/s1, Figure S1, SDS-PAGE analysis of the secreted proteins of Foc. The gel was stained with CBB to visualize total proteins. Ten μg total proteins per lane were loaded. Lane M, protein marker; lane 1, the secreted proteins from Foc1 at 7 h after banana extracts induction; lane 2, the secreted proteins from Foc1 at 11 h after banana extracts induction; lane 3, the secreted proteins from Foc4 at 7 h after banana extracts induction; lane 4, the secreted proteins from Foc4 at 11 h after banana extracts induction. Figure S2, Venn diagram analysis of the secreted proteins that overlapped between three biological replicates in Foc1 (A) and Foc4 (B). Figure S3, The amino acid length distribution of the secreted proteins in Foc1 and Foc4. Figure S4, Venn diagram analysis of the secreted proteins that overlapped between Foc1 (yellow) and Foc4 (blue). A, Total secreted proteins. B, Extracelluar classically-secreted proteins. C, Cell membrane classically secreted proteins. D, Extracelluar non-classically secreted proteins. The diagram shows the number of the secreted proteins specifically in each race as well the number in both races. Table S1, Primers used for amplification of the secreted proteins. Table S2, Results of all bioinformatic analyses carried out in this study. Table S3, The annotation of the secreted proteins used for RT-qPCR analysis.
