# Supplementary material for: In Vitro Secretome Analysis Suggests Differential Pathogenic Mechanisms between Fusarium oxysporum f. sp. cubense Race 1 and Race 4
Source: Biomolecules. 2021 Sep 12;11(9):1353. doi: 10.3390/biom11091353 (PMC8466104; doi:10.3390/biom11091353)
Supplement: Supplementary file 1 [file biomolecules-11-01353-s001.zip › Supplementary Figure S1-S4.pdf]

Supplementary Figures

In vitro secretome analysis suggests differential pathogenic mechanisms between *Fusarium oxysporum* f. sp. *cubense* race 1 and race 4

Figure S1.

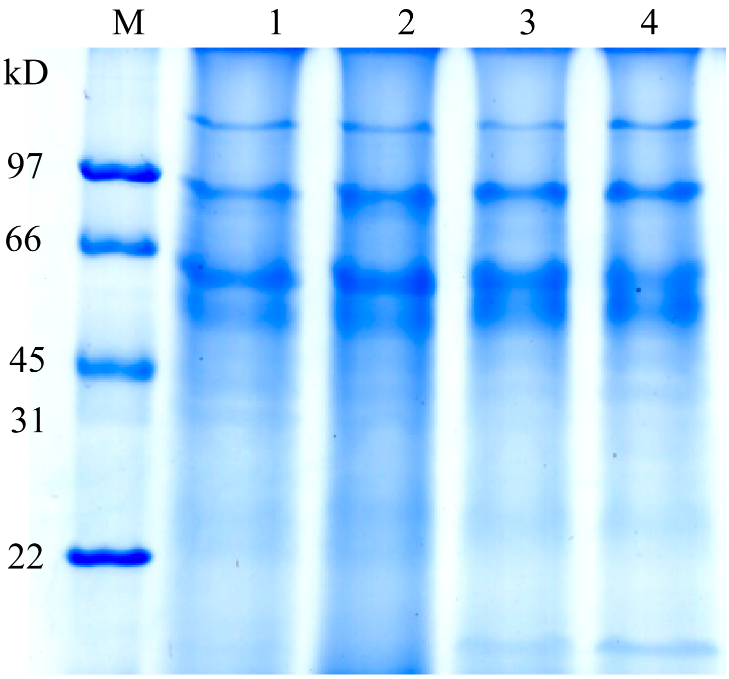

Figure S1. SDS-PAGE analysis of the secreted proteins of Foc. The gel was stained with CBB to visualize total proteins. Ten μg total proteins per lane were loaded. Lane M, protein marker; lane 1, the secreted proteins from Foc1 at 7 h after banana extracts induction; lane 2, the secreted proteins from Foc1 at 11 h after banana extracts induction; lane 3, the secreted proteins from Foc4 at 7 h after banana extracts induction; lane 4, the secreted proteins from Foc4 at 11 h after banana extracts induction.

Figure S2.

A

B

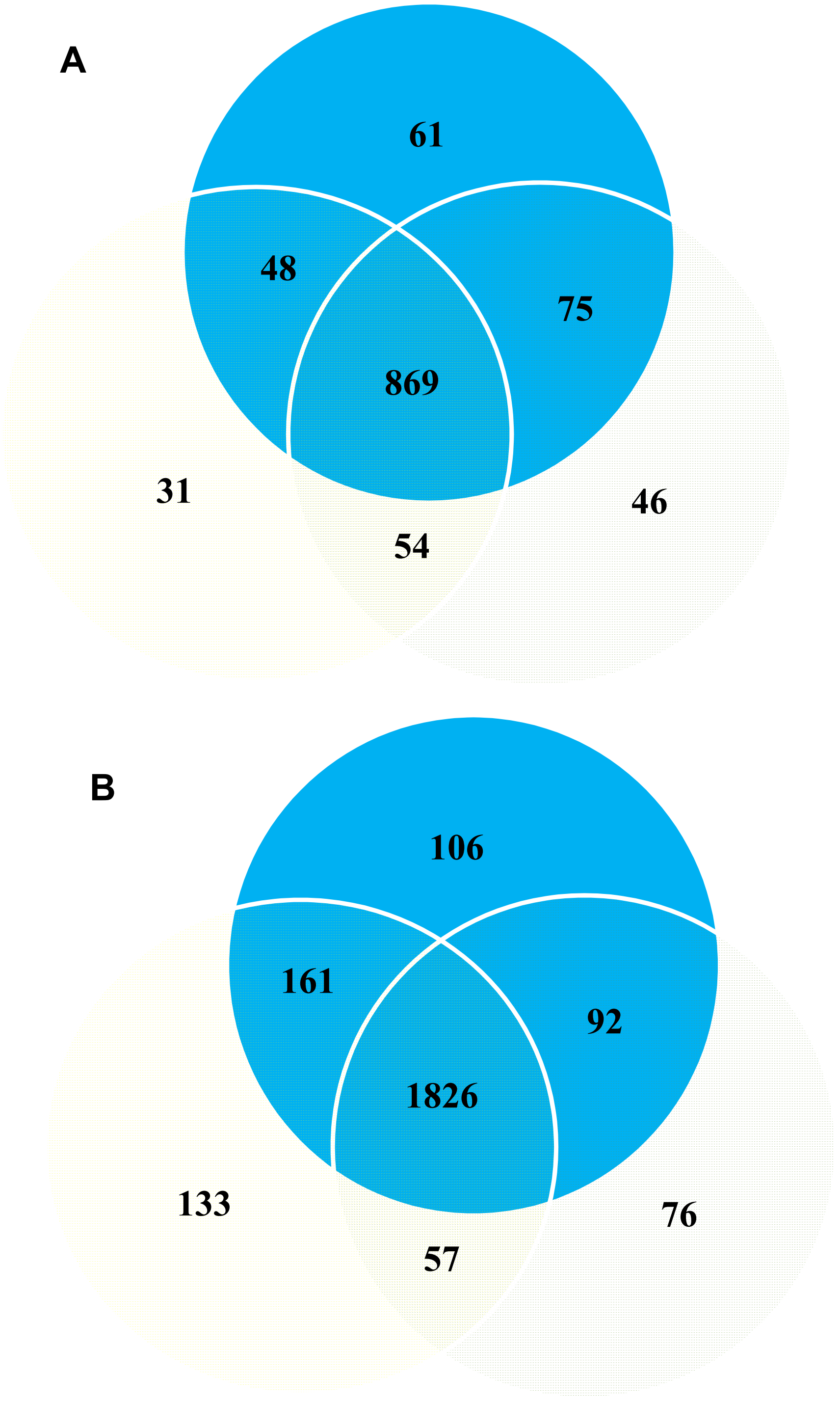

Figure S2. Venn diagram analysis of the secreted proteins that overlapped between three biological replicates in Foc1 (A) and Foc4 (B).

Figure S3

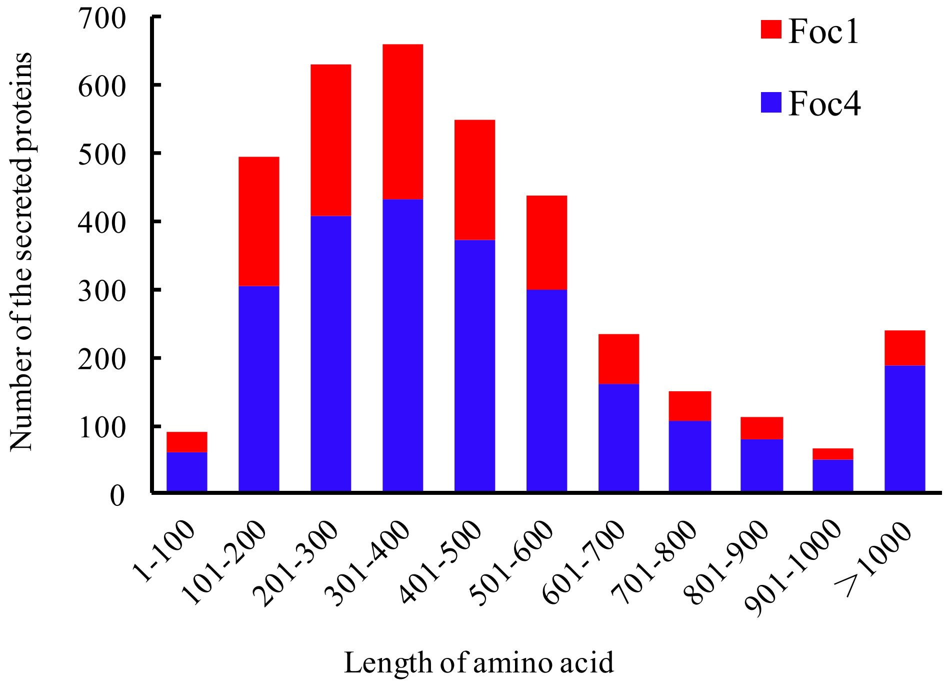

Figure S3. The amino acid length distribution of the secreted proteins in Foc1 and Foc4.

Figure S4

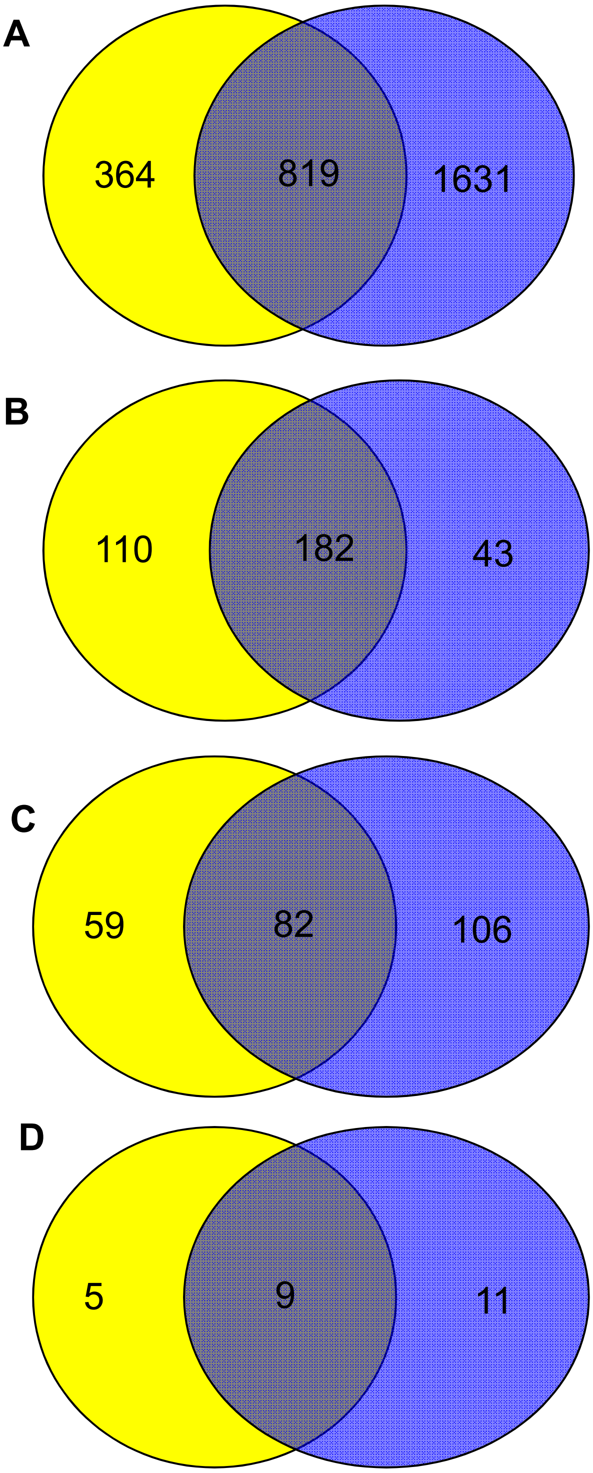

Figure S4. Venn diagram analysis of the proteins that overlapped between Foc1 (yellow) and Foc4 (blue). A, total secreted proteins. B, Extracellular classically-secreted proteins. C, Cell membrane non-classically secreted proteins. D, Extracellular non-classically-secreted proteins. The diagram shows the number of the secreted proteins specifically in each races as well the number in both races.
