# Supplementary material for: In Vitro Secretome Analysis Suggests Differential Pathogenic Mechanisms between Fusarium oxysporum f. sp. cubense Race 1 and Race 4
Source: Biomolecules. 2021 Sep 12;11(9):1353. doi: 10.3390/biom11091353 (PMC8466104; doi:10.3390/biom11091353)
Supplement: Supplementary file 1 [file biomolecules-11-01353-s001.zip › Supplementary Tables S1 and S3.pdf]

Supplementary Tables

Table S1. Gene-specific primers sequences designed for qRT-PCR.

Protein Name	Gene Name (Foc1/Foc4)	Forward Primer	Reverse Primer
Cutinase	ENH74517.1/ EXL99094.1	TGTTGTACGCTGTGATCTGCT	CACTTCTTCTACTCGCTACTGCTA
Glycosyl hydrolase family 17	ENH73451.1/ EXL90453.1	TTGACGTGCTTCTGAACATTCC	ATCACCAACGGCTTTCCCTA
Endopolygalacturonase	ENH63545.1/EXL90516.1	ATGTTGTATCCGCTGGAAGAA	TGAGCAGAACTACTATGGTGGC
Polygalacturonase	ENH75544.1/EXL99525.1	CTGTGGAAGAACCGATGTCAAA	GTGGGATGGAAACGGAGGAA
Endo-1,3(4)-beta-glucanase	ENH65756.1/EXM05404.1	TGGAGAATGTCAACGGGCAGAA	CAAACTGGACCCGCCAAACG
Alpha 1,3-glucosidase	ENH68739.1/EXM07121.1	TTGGCATTCCTGAACATA	AGCCGTAAAGCGTCATT
Trypsin	ENH63648.1/EXL97312.1	GACGGTGTTGGCGTTGAGGA	ACATCGTTGGCGGCACTTCT
SIX1	ENH66349.1/EXL90175.1	TCCCAGACGAGTGATTTGC	AGTCGCCCGACGGTTAG
Cytochrome P450 55A1	ENH66511.1/EXL92621.1	TCTAAGCCATCAGAACGA	GGTCTTGGGCGACTTTT
Peptidase A1 domain-containing protein	ENH72305.1/EXL91252.1	ATCCGCCAAGTAACCAG	CAATGCCACCAAGAATAA
Pyr_redox_2 domain-containing protein	ENH63877.1/EXM07548.1	AACCTGCCTATTTCCGCTACG	GCTGTGATCTTGGTGGTCCTG
N4-(Beta-N-acetylglucosaminyl)-L-asparaginase	ENH67070.1/EXM02659.1	TGGTCGTGGTCTTGCTGGTG	GGGCTGCTTGTGGAAGTATCG
β-Tublin	reference gene	TCAGTGCGGTAACCAAATCGG	GACGGCTCGGGGAACATACTTG

Table S3. The annotation of the secreted proteins used for qRT-PCR analysis.

Protein Name	Gene Name (Foc1/Foc4)	CAZymes Annotation	PHI Annotation
Cutinase	ENH74517.1/ EXL99094.1	CE3	PHI:407 loss_of_pathogenicity
Glycosyl hydrolase family 17	ENH73451.1/ EXL90453.1	GH17	PHI:2896 reduced_virulence
Endopolygalacturonase	ENH63545.1/EXL90516.1	GH28	PHI:2818 reduced_virulence
Polygalacturonase	ENH75544.1/EXL99525.1	GH28	PHI:2817 unaffected_pathogenicity
Endo-1,3(4)-Treta-glucanase	ENH65756.1/EXM05404.1	GH16	-
Alpha 1,3-glucosidase	ENH68739.1/EXM07121.1	GH31	PHI:6739 reduced_virulence
Trypsin	ENH63648.1/EXL97312.1	-	PHI:652 effector
SIX1	ENH66349.1/EXL90175.1	-	PHI:379 SIX1 effector
Cytochrome P450 55A1	ENH66511.1/EXL92621.1	-	PHI:6739 reduced_virulence
Peptidase A1 domain-containing protein	ENH72305.1/EXL91252.1	-	PHI:6815 unaffected_pathogenicity
Pyr_redox_2 domain-containing protein	ENH63877.1/EXM07548.1	-	-
N4-(Treta-N-acetylglucosaminyl)-L-asparaginase	ENH67070.1/EXM02659.1	-	-
